# Risk of incident fractures in individuals hospitalised due to unexplained syncope and orthostatic hypotension

**DOI:** 10.1186/s12916-021-02065-7

**Published:** 2021-08-27

**Authors:** Madeleine Johansson, Cecilia Rogmark, Richard Sutton, Artur Fedorowski, Viktor Hamrefors

**Affiliations:** 1 Department of Clinical Sciences, Clinical Research Center, Lund University, Box 50332, 20213 Malmö, Sweden; 2 Department of Cardiology, Skåne University Hospital, Malmö, Sweden; 3 Department of Orthopaedics, Skåne University Hospital, Lund University, Malmö, Sweden; 4 National Heart and Lung Institute, Imperial College, Hammersmith Hospital Campus, London, UK; 5 Department of Internal Medicine, Skåne University Hospital, Malmö, Sweden

**Keywords:** Syncope, Orthostatic hypotension, Fractures, Falls, Prevention

## Abstract

**Background:**

Impaired orthostatic blood pressure response and syncope confer a high risk of falls and trauma. The relationship between a history of unexplained syncope and orthostatic hypotension (OH) with subsequent fractures, however, has not been thoroughly examined. In this study, we aimed to investigate the relationship between previous hospital admissions due to unexplained syncope and OH and incident fractures in a middle-aged population.

**Methods:**

We analysed a large population-based prospective cohort of 30,399 middle-aged individuals (age, 57.5 ± 7.6; women, 60.2%). We included individuals hospitalised due to unexplained syncope or OH as the main diagnosis. Multivariable-adjusted Cox regression analysis was applied to assess the impact of unexplained syncope and OH hospitalisations on subsequent incident fractures.

**Results:**

During a follow-up period of 17.8 + 6.5 years, 8201 (27%) subjects suffered incident fractures. The mean time from baseline and first admission for syncope (*n* = 493) or OH (*n* = 406) was 12.6 ± 4.2 years, and the mean age of the first hospitalisation was 74.6 ± 7.4 years. Individuals with incident fractures were older, more likely to be women, and had lower BMI, higher prevalence of prevalent fractures, and family history of fractures. Multivariable-adjusted Cox regression showed an increased risk of incident fractures following hospitalisations due to unexplained syncope (HR 1.20; 95% CI 1.02–1.40; *p* = 0.025) and OH (HR 1.42; 95% CI 1.21–1.66; *p* < 0.001) compared with unaffected individuals.

**Conclusions:**

Individuals hospitalised due to unexplained syncope and orthostatic hypotension have an increased risk of subsequent fractures. Our findings suggest that such individuals should be clinically assessed for their syncope aetiology, with preventative measures aimed at fall and fracture risk assessment and management.

**Supplementary Information:**

The online version contains supplementary material available at 10.1186/s12916-021-02065-7.

## Background

Syncope is a transient loss of consciousness due to cerebral hypoperfusion and is characterised by a rapid onset, short duration, and spontaneous complete recovery. The causes of syncope may be broadly divided into reflex syncope, orthostatic hypotension (OH), and primary cardiac syncope [1]. Orthostatic hypotension is the second most common aetiology of syncope, occurring in approximately 15% of syncope presentations [2, 3].

The overlap between reflex syncope, orthostatic hypotension, and falls is becoming increasingly acknowledged [4]. Fractures resulting from falls constitute a leading public health concern, since they are associated with extensive human and socio-economic impact, morbidity, mortality, and costs [5]. Moreover, the number of fractures is expected to increase as the population of older individuals increases [6].

Unexplained falls may mask a diagnosis of syncope in nearly 50% of cases [7]. Yet, establishing the cause of a fall remains difficult, and almost 30% of older individuals have difficulties recalling a minor fall after 3 months [8]. In this study, we aimed to investigate the relationship of hospital admissions due to unexplained syncope and orthostatic hypotension with subsequent fractures in a large Swedish middle-aged population followed over nearly 18 years.

## Methods

### Study population and design

The Malmö Diet and Cancer (MDC) study is a large prospective population-based study launched in the 1990s. A total of 30,446 participants were included (41% participation rate; age, 57.5 ± 7.6; women, 60.2%) and attended the baseline examination between 1991 and 1996. Details of the MDC study have been described elsewhere [9].

**Fig. 1:**
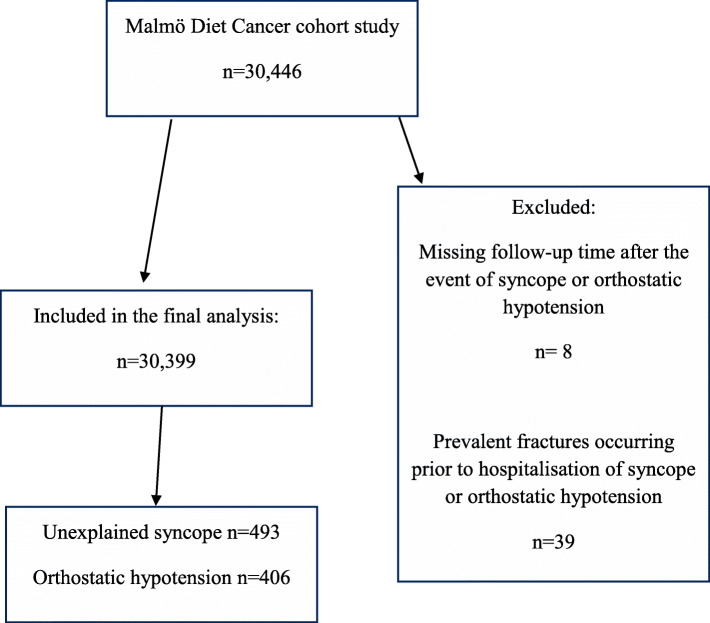
Flowchart of participant selection

In the current study, we excluded individuals with fractures that had occurred prior to hospitalisation for syncope or orthostatic hypotension (*n* = 39), and individuals with missing follow-up time data (*n* = 8), leaving 30,399 individuals that were included in the final analysis. Of these, 8201 subjects had incident fractures occurring after baseline examination (Fig. 1). One of the primary outcomes were incident fractures occurring after hospital admissions for unexplained syncope or orthostatic hypotension compared with the individuals without a history of unexplained syncope or orthostatic hypotension hospitalisation.

### Data on hospitalisation and incident fractures

Data on hospitalisation for unexplained syncope, orthostatic hypotension, and incident fractures were retrieved from the Swedish National Patient Register [10], which includes complete coverage of public in-patient care since 1987 and out-patient care since 2001. Thus, fractures occurring prior to 2001 were only included if they resulted in hospital admission.

Follow-up data on hospitalisations was available through December 31, 2016. Follow-up time was defined as the time from baseline examination until the first incident fracture, death, emigration, or end of follow-up on December 31, 2016, whichever occurred first.

Unexplained syncope and orthostatic hypotension were defined according to the International Classification of Diseases 9th and 10th Revisions (ICD-9 and ICD-10), based on the primary or main secondary discharge diagnoses: 780.2, R550.9; 458 and I951.

Fractures were defined according to the following ICD-10 codes: spine and thorax (S12.x, S22.x, S32.x); arms, shoulders, and hands (S42.x, S52.x, S62.x); and pelvis (S32.x), hips, femur, and lower leg (S72.x, S82.x). In addition, ICD codes signalling spontaneous fractures and peri-implant fractures were also sought (M48.4 non-traumatic vertebral fracture, M84.3 stress fracture, and M96.6 peri-implant fractures; however, M97 is not used in Sweden). A total of 503 fractures occurring prior to 1997 and coded by corresponding ICD-9 codes were included.

### Baseline variables and definition of clinical characteristics

The following variables were obtained at baseline examination: age (*n* = 30,393), sex (*n* = 30,399), body mass index (BMI) (*n* = 30,341), systolic blood pressure (SBP) (*n* = 30,344), and diastolic blood pressure (DBP) (*n* = 30,341). Blood pressure was measured using a sphygmomanometer and properly sized right arm cuff after a 10-min rest in the supine position. The following variables were self-reported in the questionnaire: current smoking, defined as regular or occasional smoking (*n* = 28,523), and family history of fracture, defined as any fracture in mother/father/sibling after 50 years of age (*n* = 30,399). Since bone density data is unavailable in the MDC cohort, we used data on prevalent fracture and family history of fracture as a proxy of poor bone health. Data on fractures was collected from the Swedish National Patient Register.

### Statistical analyses

Continuous data are shown as mean ± standard deviation, whereas frequencies are used to describe categorical data. Continuous variables were compared using the paired Student’s *t*-test. All continuous variables that were used in the current study were normally distributed, as assessed from visual inspection of histograms. Paired and multiple proportions were compared using Pearson’s chi-square test.

Two Cox regression models were constructed to assess the association of unexplained syncope and orthostatic hypotension hospitalisations with subsequent fractures. The basic model included age and sex, whereas the multivariable model included age, sex, prevalent fractures, family history of fractures, BMI, and current smoking.

The long-term cumulative incidence of fractures was stratified according to the presence or absence of hospital admission for unexplained syncope and orthostatic hypotension and calculated using the Kaplan-Meier method with quantification using the log-rank test.

All statistical analyses were performed in IBM SPSS Statistics 27 (IBM Corporation, Armonk, NY, USA).

## Results

During the follow-up period (mean time 17.8 + 6.5 years), a total of 8201 subjects (27%) suffered at least one fracture. Individuals with incident fractures were older, more likely to be women, and had lower BMI, higher prevalence of prevalent fractures, and family history of fractures. Baseline characteristics of participants are shown in Table 1. Baseline characteristics of the study population stratified according to hospitalisations due to orthostatic hypotension and unexplained syncope can be found in Supplementary Table S1.

**Table 1:** Baseline characteristics of the study population (*n* = 30,399) stratified according to the presence of incident fractures

	Incident fractures (***n*** = 8201)	No incident fractures (***n*** = 22,198)
Age (years ± SD)	59.0 ± 7.8	57.0 ± 7.5
Sex, female (%)	73.1	55.4
BMI (kg/m^2^ ± SD)	25.6 ± 4.1	25.9 ± 4.0
Current smoking (%)	27.5	28.6
Prevalent fracture (%)	4.6	2.4
Family history of fracture (%)	21.3	15.9
Unexplained syncope hospitalisations (%)	2.0	1.5
Orthostatic hypotension hospitalisations (%)	2.0	1.1
SBP (mmHg ± SD)	141.8 ± 20.1	140.9 ± 20.0
DBP (mmHg ± SD)	85.3 ± 9.8	85.7 ± 10.1

*p*-values for differences all < 0.001

Abbreviations: *BMI* body mass index; *DBP* diastolic blood pressure; *SBP* systolic blood pressure; *SD* standard deviation

The mean time between baseline and the first admission for syncope and orthostatic hypotension was 12.6 ± 4.2 years, while the mean age at the time of the first hospitalisation was 74.6 ± 7.4 years. In total, 493 and 406 individuals were hospitalised due to unexplained syncope and orthostatic hypotension, respectively. In the two groups, 49.9% and 50.2% were female, respectively. The mean age at baseline of individuals with unexplained syncope was 61.5 ± 7.1 years, and for orthostatic hypotension individuals, 62.6 ± 6.6 years.

**Fig. 2:**
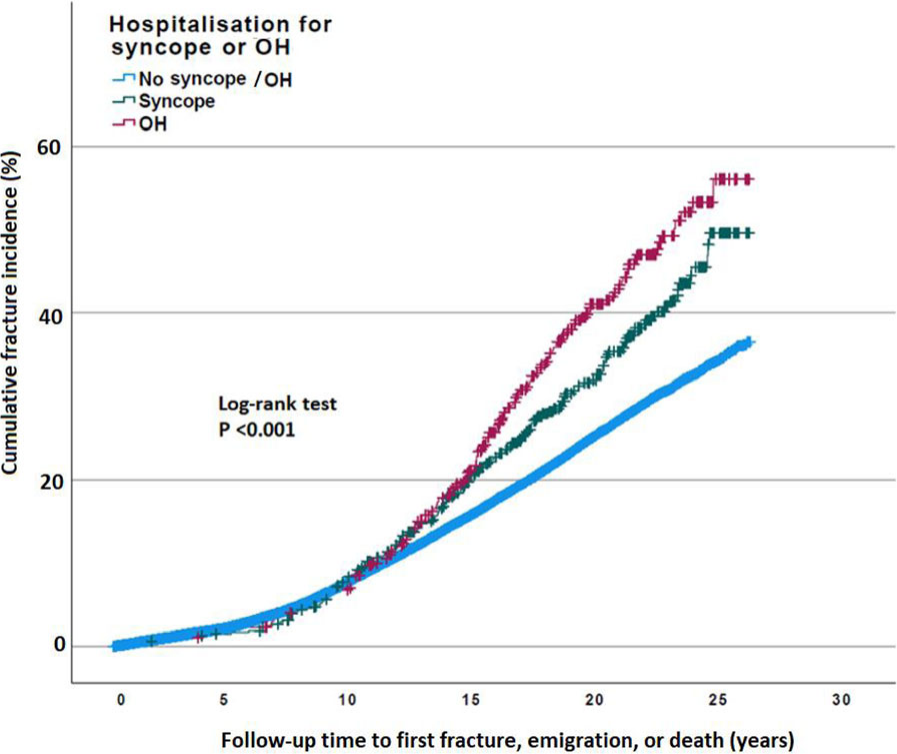
The long-term cumulative incidence of fractures stratified according to previous hospitalisations due to unexplained syncope and orthostatic hypotension. Kaplan-Meier curves depicting the long-term cumulative incidence of fractures stratified according to previous hospitalisations resulting from unexplained syncope (green, *n* = 493) and orthostatic hypotension (OH; red, *n* = 406) among 30,399 middle-aged individuals (mean age, 57.5 ± 7.6; women, 60.2%). A significantly higher incidence of fractures in individuals previously hospitalised due to unexplained syncope and orthostatic hypotension (log-rank test: *p* < 0.001) was observed with both survival curves diverging from the control group around 12 years from inclusion, i.e. the median time between baseline and syncope or orthostatic hypotension, and a second diversion between unexplained syncope and orthostatic hypotension was observed at 15 years

Individuals previously hospitalised due to unexplained syncope and orthostatic hypotension had a higher incidence of fractures (log-rank test: *p* < 0.001). The survival curves for both groups diverged from the control group around 12 years from inclusion, i.e. the median time between baseline and syncope or orthostatic hypotension. A second diversion between unexplained syncope and orthostatic hypotension was observed at 15 years (Fig. 2).

In the basic Cox regression model, there was an increased risk of incident fractures following hospitalisations due to unexplained syncope (hazard ratio (HR) 1.17; 95% confidence interval (CI) 1.01–1.37; *p* = 0.042) and orthostatic hypotension (HR 1.41; 95% CI 1.21–1.65; *p* < 0.001). The multivariable-adjusted model showed similar results, with an increased risk of incident fractures following hospitalisations due to unexplained syncope (HR 1.20; 95% CI 1.02–1.40; *p* = 0.025) and orthostatic hypotension (HR 1.42; 95% CI 1.21–1.66; *p* < 0.001) (Table 2). The proportion of subjects who suffered subsequent fracture did not differ significantly between subjects with only one hospitalisation (35.4%) compared with two or more hospitalisations (40.5%; *p* = 0.141). Moreover, in the basic Cox regression model, the effect size was only slightly higher in subjects with two or more episodes (HR 1.32; 95% CI 1.10–1.59) compared with subjects with only one episode (HR 1.26; 95% CI 1.10–1.44).

**Table 2:** Risk factors for incident fractures

	Basic model			Multivariate model		
	HR	95% CI	***p***-value	HR	95% CI	***p***-value
Unexplained syncope hospitalisations	1.17	1.01–1.37	0.042	1.20	1.02–1.40	0.025
OH hospitalisations	1.41	1.21–1.65	< 0.001	1.42	1.21–1.66	< 0.001
Age (per year)	1.06	1.05–1.06	< 0.001	1.06	1.05–1.06	< 0.001
Sex (female)	1.91	1.82–2.01	< 0.001	1.89	1.80–1.99	< 0.001
BMI (per kg/m^2^)	0.98	0.98–0.99	< 0.001	0.98	0.98–0.99	< 0.001
Current smoking	1.22	1.16–1.28	< 0.001	1.20	1.14–1.26	< 0.001
Prevalent fractures	1.83	1.65–2.02	< 0.001	1.78	1.59–1.98	< 0.001
Family history of fractures	1.16	1.10–1.22	< 0.001	1.17	1.11–1.23	< 0.001

Basic and multivariable Cox regression analysis of the relationship between risk factors and incident fractures. Basic model adjusted for age and sex. Multivariable model adjusted for age, sex, BMI, current smoking, prevalent fractures, and family history of fractures

Abbreviations: *BMI* body mass index; *CI* confidence interval; *HR* hazard ratio; *OH* orthostatic hypotension

## Discussion

In this large prospective, population-based, observational study, the risk of incident fractures following hospitalisations due to unexplained syncope and orthostatic hypotension was increased by 20% and 42%, respectively. Thus, the effect size of unexplained syncope and orthostatic hypotension on the risk of fractures in our cohort is similar to that of several established risk factors for fractures, such as smoking and family history of fractures [11].

### Strengths and weaknesses of the study

To our knowledge, this is the first prospective observational study investigating the association between incident fractures following hospitalisations due to unexplained syncope and orthostatic hypotension. The strength of this study is the use of a large population-based cohort with over 30,000 middle-aged individuals enrolled and minimal loss to follow-up. Moreover, data on hospitalisations due to syncope and orthostatic hypotension were retrieved from a national register with complete coverage of public healthcare in Sweden, making our findings reliable and robust. Previous studies have focused on examining the association between unexplained falls in individuals with syncope and orthostatic hypotension [4, 7, 12]. Our findings provide additional knowledge related to the importance of fracture prevention in the population. The mortality in the MDC cohort has been explored in several previous studies. For example, a recent study showed that the cumulative survival after 10 years is around 90% [13]. A previous study from our research group concluded that syncope and OH increased the risk of death in the MDC cohort [14]. Thus, based on the assumption of competing risk, we anticipate that this would underestimate rather than overestimate our current associations.

Our study has some limitations that need to be addressed. The number of subjects that was excluded due to incident fracture before hospitalisation for syncope or OH was small. Two of the baseline variables (current smoking and family history of fracture) were self-reported in the questionnaire, suggesting that there may be some response bias. Also, data on bone density was unavailable in the cohort; consequently, we used prevalent fracture and family history of fracture as a proxy of poor bone health. Data on serum vitamin D levels, vitamin D supplementation, dementia, and baseline resting heart rate was unavailable in the cohort, which otherwise would have been of interest to analyse. One explanation for the observed lower hazard ratio in the syncope groups compared with OH hospitalisation may be that individuals with OH were older and may have more co-morbidities. Although therapy for syncope is available once the arrhythmic or hypotensive aetiology is established, patients with syncope and OH may demonstrate treatment failure due to comorbidities that are commonly observed at this age, e.g. cognitive and physical impairment. Unfortunately, we have no data available on intervention against syncope hospitalisation.

There was a considerable lag between baseline and the exposure to OH/syncope. In the current study, we excluded individuals with fractures that had occurred prior to hospitalisation for syncope or orthostatic hypotension. However, there were only 39 subjects that had fractures prior to possible hospitalisation. We observed no significant difference in time between baseline and incident fracture for syncope/OH and no syncope/OH group (*p* = 0.57), indicating that hospitalisations did not accelerate fractures.

The exclusion of subjects with fractures prior to hospitalisation (*n* = 39) was naturally restricted to the hospitalisation group. The vast majority of fractures in subjects with syncope or OH hospitalisations occurred after hospitalisation after which 332 (36.9%) of the subjects had a fracture. As a comparison, after 12 years (i.e. the mean follow-up from baseline to the first syncope/OH hospitalisation), 4663 (15.3%) subjects in the non-hospitalisation group experienced fractures.

### The current results in relation to similar studies

To the best of our knowledge, our study is the first to provide effect sizes of unexplained syncope and orthostatic hypotension in terms of incident fracture risk in the population. A correlation between cardiovascular instability, hypotension, and fractures has been previously demonstrated, suggesting that fractures due to low-energy trauma (falling from a standing height or less) may serve as a valid surrogate marker for syncope and blood pressure instability [15, 16]. In a previous study of individuals aged 50 and above, the prospective risk of fracture associated with delayed BP recovery was 7% [17]. As only 5–10% of falls lead to a fracture [18], we believe our study, examining incident fractures as an outcome, provides additional clinically relevant information, since previous studies on syncope and orthostatic hypotension have only investigated falls as an outcome. Thus, studying the risk of fracture due to falls is highly relevant, as falls are the single strongest risk factor for fracture, suggesting that the focus in fracture prevention may benefit from a shift from osteoporosis to falls [19].

In 90% of cases in the elderly, femoral fractures are preceded by a fall [20]. Unexplained falls commonly lead to hospitalisations and increased healthcare costs [21], and over 33% of falls at orthopaedic wards are unexplained [22]. A previous study found that 25% of adults aged above 50 years presenting to the emergency department due to a fall had symptoms suggestive of syncope or an unexplained fall [4]. In addition, nearly 20% of individuals above 70 years with orthostatic hypotension presented as falls, signalling that orthostatic hypotension is also an important independent risk factor for future falls [23].

A syncopal mechanism may underlie an unexplained fall [1]. Our results emphasise the importance of fracture risk evaluation in individuals that have been hospitalised for syncope or orthostatic hypotension, since substantial clinical consequences result from overlooked and missed diagnoses of syncope, that could otherwise have been addressed by adequate management of the underlying syncopal cause. Systolic blood pressure tends to increase over time but decreases in later life [24]. One of the underlying explanations may be arterial stiffness. Arterial stiffness is independently associated with orthostatic hypotension [25]. An increase in peripheral vascular resistance at rest is not routinely observed in healthy older persons, but is often associated with increased stiffness of central elastic arteries, as hallmarks of ageing effects on the vasculature [26]. Impaired stretching of the carotid arterial wall close to the baroreceptor due to arterial stiffness and superimposed atherosclerosis may lead to impaired baroreceptor function in response to change of body position, thus being a potential marker of orthostatic reactions [26].

### Meaning of the study and clinical implication

Our study results indicate that syncope and orthostatic hypotension are two factors that may be overlooked during fracture prevention. During the last decade, falls clinics have been implemented on a small scale [27]. Falls clinics and syncope units constitute an important tool in the management of unexplained falls, syncope, and orthostatic hypotension [1]. Although large randomised studies are currently lacking, our findings support the importance of implementing multidisciplinary teams with falls and syncope specialists. In addition, general preventive efforts against fracture and fall should be undertaken, for example, restrictive use of medications known to increase fall risk (e.g. anti-hypertensive and psychotropic drugs), implementation of home hazard reduction (e.g. rails, nonslip mats, the use of shoes with nonslip soles), and tailored exercise programmes improving mobility and quality of life [19, 20, 28–31]. Lifestyle changes with proper nutrition are also essential, since nutritional status is an independent predictor of falls in older people living in the community [32].

### Unanswered questions and future research

The observed increased risk of incident fractures in individuals with orthostatic hypotension compared with syncope in the present study may result from possible concomitant diseases such as Parkinson’s disease or dementia, which are well-established factors associated with postural instability and gait dysfunction that may lead to an increased risk for falls and injuries [33–35]. Although such investigations are beyond the scope of this paper, future research warrants assessment of the level and potential role of dementia in patients with concomitant orthostatic hypotension or unexplained syncope who are at high risk of incident fractures.

## Conclusion

Individuals hospitalised due to unexplained syncope and orthostatic hypotension have a markedly increased risk of subsequent fractures. This population-based study is the first to provide effect sizes of unexplained syncope and orthostatic hypotension in terms of incident fracture risk in the population. Our findings suggest that such individuals should be clinically assessed for their syncope aetiology, and preventative measures aimed at fall and fracture risk assessment, and management.

## Supplementary Information

**Additional file 1: Table S1.** Baseline characteristics of the study population (*n*=30,399) stratified according to hospitalisations due to orthostatic hypotension and unexplained syncope. *P*-values for differences all <0.001. Abbreviations: BMI, body mass index; DBP, diastolic blood pressure; SBP, systolic blood pressure; SD, standard deviation.

## Data Availability

Data are available upon reasonable request to the corresponding author.

## Abbreviations

BMI: Body mass index
DBP: Diastolic blood pressure
OH: Orthostatic hypotension
SBP: Systolic blood pressure
