# Supplementary material for: Risk of incident fractures in individuals hospitalised due to unexplained syncope and orthostatic hypotension
Source: BMC Med. 2021 Aug 27;19:188. doi: 10.1186/s12916-021-02065-7 (PMC8394111; doi:10.1186/s12916-021-02065-7)
Supplement: Supplementary file 1 — Additional file 1: Table S1. Baseline characteristics of the study population (n=30,399) stratified according to hospitalisations due to orthostatic hypotension and unexplained syncope. P-values for differences all <0.001. Abbreviations: BMI, body mass index; DBP, diastolic blood pressure; SBP, systolic blood pressure; SD, standard deviation. [file 12916_2021_2065_MOESM1_ESM.docx]

**Supplementary tables**

**Table S1. Baseline characteristics of the study population (n=30,399) stratified according to hospitalisations due to orthostatic hypotension and unexplained syncope.**

|  | **Unexplained syncope**  **(n=493)** | **Orthostatic hypotension**  **(n=406)** | **No hospitalization**  **(n=29,500)** |
| --- | --- | --- | --- |
| Age, (years ± SD) | 61.6 ± 7.0 | 62.5 ± 6.7 | 57.4 ± 7.6 |
| Sex, female, (%) | 49.9 | 50.2 | 60.5 |
| BMI, (kg/m^2^ ± SD) | 26.8 ± 4.3 | 25.9 ± 4.3 | 25.8 ± 4.0 |
| Current smoking, (%) | 25.1 | 24.7 | 28.4 |
| Previous fracture, (%) | 3.4 | 2.7 | 3.0 |
| Family history of fracture, (%) | 17.2 | 16.0 | 17.4 |
| Incident fractures, (%) | 34.1 | 40.4 | 26.7 |
| Anti-hypertensive treatment, (%) | 27.6 | 23.4 | 17.1 |
| SBP, (mmHg ± SD) | 148.4 ± 20.9 | 146.4 ± 21.3 | 140.9 ± 20.0 |
| DBP, (mmHg ± SD) | 87.6 ± 10.1 | 62.5 ± 6.7 | 85.6 ± 10.0 |

*P-values for differences all <0.001.
Abbreviations: BMI, body mass index; DBP, diastolic blood pressure; SBP, systolic blood pressure; SD, standard deviation*
